# Field Trial Performance of Herculex XTRA (Cry34Ab1/Cry35Ab1) and SmartStax (Cry34Ab1/Cry35Ab1 + Cry3Bb1) Hybrids and Soil Insecticides Against Western and Northern Corn Rootworms (Coleoptera: Chrysomelidae)

**DOI:** 10.1093/jee/tox099

**Published:** 2017-04-18

**Authors:** K. D. Johnson, L. A. Campbell, M. D. Lepping, D. M. Rule

**Affiliations:** Dow AgroSciences, 9330 Zionsville Rd., Indianapolis, IN 46268 (kdjohnson@dow.com; lacampbell@dow.com; mdlepping@dow.com; DDRule@dow.com); 1 Corresponding author, e-mail: kdjohnson@dow.com

**Keywords:** SmartStax, Cry34Ab1/Cry35Ab1, Cry3Bb1, western corn rootworm, northern corn rootworm

## Abstract

Western corn rootworm, *Diabrotica virgifera virgifera* LeConte (Coleoptera: Chrysomelidae), and northern corn rootworm, *Diabrotica barberi* Smith and Lawrence (Coleoptera: Chrysomelidae), are important insect pests in corn, *Zea mays* L. For more than a decade, growers have been using transgenic plants expressing proteins from the bacterium *Bacillus thuringiensis* (Bt) to protect corn roots from feeding. In 2011, western corn rootworm populations were reported to have developed resistance to Bt hybrids expressing Cry3Bb1 and later found to be cross-resistant to hybrids expressing mCry3A and eCry3.1Ab. The identification of resistance to Cry3 (Cry3Bb1, mCry3A, and eCry3.1Ab) hybrids led to concerns about durability and efficacy of products with single traits and of products containing a pyramid of a Cry3 protein and the binary Bt proteins Cry34Ab1 and Cry35Ab1. From 2012 to 2014, 43 field trials were conducted across the central United States to estimate root protection provided by plants expressing Cry34Ab1/Cry35Ab1 alone (Herculex RW) or pyramided with Cry3Bb1 (SmartStax). These technologies were evaluated with and without soil-applied insecticides to determine if additional management measures provided benefit where Cry3 performance was reduced. Trials were categorized for analysis based on rootworm damage levels on Cry3-expressing hybrids and rootworm feeding pressure within each trial. Across scenarios, Cry34Ab1/Cry35Ab1 hybrids provided excellent root protection. Pyramided traits provided greater root and yield protection than non-Bt plus a soil-applied insecticide, and only in trials where larval feeding pressure exceeded two nodes of damage did Cry34Ab1/Cry35Ab1 single-trait hybrids and pyramided hybrids show greater root protection from the addition of soil-applied insecticides.

Western corn rootworm, *Diabrotica virgifera virgifera* LeConte, and northern corn rootworm, *Diabroticabarberi* Smith and Lawrence (Coleoptera: Chrysomelidae), are significant pests of corn, *Zea mays* L., in the United States. The larval stage of these species feed on corn roots, causing root pruning and yield loss when at damaging levels. Root pruning causes yield loss of up to 17% per node of root consumed (Tinsley et al. 2013). This yield loss is the result of inhibiting the plant’s ability to take up water and nutrients from the soil, leading to decreased photosynthetic activity (Spike and Tollefson 1989, Sutter and Gustin 1990, Spike and Tollefson 1991, Dunn and Frommelt 1998). Severe root pruning may also lead to plant lodging, which may cause losses due to a reduction in light interception, and additional losses due to the difficulty of harvesting lodged plants (Sutter and Gustin 1990).

Soil-applied insecticides have been the historical root protection tactic of corn rootworm management since the 1950s for continuous corn production systems (Gray et al. 1992). However, the efficacy of soil-applied insecticides may be influenced by many factors, including planting date, soil moisture, and off-target movement due to wind conditions (Sutter et al. 1989, Bergman et al. 1991, Gray et al. 1992).

Growers have had access to genetically modified corn hybrids producing transgenic proteins toxic to corn rootworms since 2003 (Vaughn et al. 2005, U.S. EPA 2016). Currently deployed proteins are derived from the soil bacterium *Bacillus thuringiensis* Berliner (Bt). Bt research has led to a variety of rootworm-active traits, which have emerged as an important management tool (U.S. EPA 2011, Keweshan et al. 2015). Herculex RW Insect Protection, registered by the U.S. Environmental Protection Agency (EPA) in 2005 (event DAS-59122-7, Dow AgroSciences LLC, Indianapolis, IN, and DuPont Pioneer, Johnston, IA), expresses two Bt proteins (Cry34Ab1 and Cry35Ab1) that form a binary toxin and reduce rootworm growth and survival (U.S. EPA 2016). In 2010, SmartStax technology (Dow AgroSciences and Monsanto Co, St. Louis, MO) was registered with the U.S. EPA for rootworm and lepidopteran control. SmartStax combines DAS-59122-7 (Cry34Ab1/Cry35Ab1) with event MON 88017 (which produces the Cry3Bb1 Bt protein) to provide two modes of action against susceptible corn rootworm populations.

Pyramided hybrids, such as SmartStax, which expresses Cry34Ab1/Cry35Ab1 and Cry3Bb1 proteins, have delivered excellent root protection against both northern and western corn rootworm under field conditions (Prasifka et al. 2013, Head et al. 2014). In 2011, Gassmann et al. (2011) documented western corn rootworm field populations resistant to hybrids expressing MON 88017 (Cry3Bb1). In 2014, cross-resistance between Cry3Bb1-expressing corn and MIR604 (mCry3A, Syngenta Crop Protection LLC, Greensboro, NC) was reported (Gassmann et al. 2014). Jakka et al. (2016) documented further cross-resistance for plants containing MIR5307 (eCry3.1Ab, Syngenta Crop Protection LLC), including pyramids of two Cry3 proteins (eCry3.1Ab1 and mCry3A). However, western corn rootworm populations resistant to corn hybrids expressing Cry3Bb1, mCry3A, or eCry3.1Ab have not been shown to be cross-resistant to hybrids expressing Cry34Ab1/Cry35Ab1 (Gassmann et al. 2011, Gassmann et al. 2014, Jakka et. al 2016). The possibility exists that the reduced efficacy of Cry3 traits could affect the efficacy and durability of Cry34Ab1/Cry35Ab1 in pyramided technologies such as SmartStax against some populations of western corn rootworm. For additional root protection against the perceived threat, corn growers began to consider adding prophylactic soil insecticides to single trait and pyramided trait technologies. Petzold-Maxwell et al. (2013) found few benefits from adding a soil insecticide to trait-protected corn when the crop is exposed to Bt-susceptible populations of corn rootworm. Tinsley et al. (2016) found a significant trend where soil insecticides provided additional root protection in Bt-susceptible populations as compared to Bt single and pyramided traits; however, the magnitude of the difference indicated that even at high pressure, producers were unlikely to experience a positive economic return.

Objectives of this research were to evaluate the root injury and yield protection offered by corn hybrids expressing insecticidal proteins (Cry34Ab1/Cry35Ab1 and Cry34Ab1/Cry35Ab1 + Cry3Bb1). The secondary goal was to evaluate the contribution of soil insecticide in fields with either a history of unexpected root injury to Cry3-expressing hybrids or fields in which Cry3 traits did not provide adequate root protection during the course of study. The results from this extensive field study will aid in the understanding product performance and in making grower recommendations for protection against corn rootworm populations that do or do not cause unexpected root injury to Cry3-expressing Bt corn in a field setting.

## Materials and Methods

Between 2012 and 2014, studies comparing Bt hybrid root injury to non-Bt root injury with and without soil insecticide were established in 31 unique sites across the U.S. Corn Belt (Table 1). Bt corn hybrids with location-adapted genetics and appropriate relative maturity were planted along with near-isogenic non-Bt hybrids (control) in each trial. Treatments are summarized in Table 2, and included a non-Bt control (NK603), Herculex XTRA (TC1507 × DAS-59122-7), and SmartStax (MON 89034 × MON 88017 × TC1507 × DAS-59122-7) hybrid each with and without the pyrethroid soil-applied insecticide (SAI) tefluthrin (Force 3G, T-band application at 185 g active ingredient/ha, Syngenta Crop Protection LLC). Each corn hybrid also contained herbicide tolerance traits. Events NK603 and MON 88017 confer tolerance to glyphosate (Monsanto Co.), and events TC1507 and DAS-59122-7 confer tolerance to glufosinate-ammonium herbicides (Bayer CropScience, Research Triangle Park, NC). Treatments containing the TC1507 event (HerculexXTRA Insect Protection and SmartStax technology) also express Cry1F, and treatments containing event MON 89034 (SmartStax) also express Cry1A.105 and Cry2Ab2 for control of lepidopteran pests. No lepidopteran pressure was observed at any of the trial locations.

**Table 1:** Trial locations, corn hybrid relative maturity, number of plot replicates, infestation type, injury to Cry3Bb1-expressing corn, feeding pressure category, and yield data collections

Year	Location	RMt^a^	Reps	Infestation source^b^	Injury level to Cry3^c^-expressing corn roots	Feeding pressure^d^	Yield^e^
2012	Crawfordsville, IA	106	4	TC	Normal	High	Y
	Nashua, IA	106	4	TC	Unexpected	High	Y
	Dekalb, IL	106	4	TC	Unexpected	High	Y
	Homer, IL	115	4	TC + AI 800	Normal	Moderate	N
	Monmouth, IL	115	4	TC	Normal	Low	Y
	Frankfort, IN	115	3	TC	Normal	Low	Y
	Fowler, IN	115	4	TC + AI 800	Normal	Low	Y
	Colman, SD	96	4	TC	Normal	High	Y
	Milbank, SD	96	4	TC	Normal	High	Y
	Hills, MN	96	4	TC	Unexpected	High	Y
	Harmony, MN	96	4	TC	Normal	Low	Y
	Higginsville, MO	115	4	TC	Normal	Low	Y
	Caroll, NE	106	4	TC	Normal	High	Y
	York, NE	106	3	TC	Normal	Low	Y
2013	Clinton, IA	115	4	TC + AI 400	Unexpected	Low	Y
	Gildden, IA	106	4	TC	Unexpected	Moderate	N
	Nashua, IA	106	4	TC	Unexpected	High	Y
	Rudd, IA	106	4	TC	Unexpected	High	Y
	Walcott, IA	106	4	TC	Normal	Moderate	Y
	Dekalb, IL	106	4	TC	Normal	Moderate	Y
	Lexington, IL	115	4	TC + AI 400	Normal	Moderate	Y
	Prophetstown, IL	115	4	TC	Unexpected	Low	Y
	Fowler, IN	115	4	TC + AI 400	Normal	Moderate	Y
	Peterson, MN	96	4	TC	Unexpected	Moderate	Y
	Rochester, MN	96	4	TC	Unexpected	Moderate	Y
	Springfield, MN	96	4	TC	Unexpected	Moderate	Y
	Beemer, NE	106	4	TC	Normal	Low	N
	Beemer, NE	106	4	TC	Normal	Low	N
	Scottsbluff, NE	96	4	TC	Normal	High	N
	York, NE	96	3	TC	Normal	Moderate	Y
	Colman, SD	96	4	TC	Unexpected	Moderate	Y
2014	Charles City, IA	106	4	TC	Normal	Low	Y
	Clinton, IA	115	4	TC + AI 400	Unexpected	Low	Y
	Rudd, IA	106	4	TC	Unexpected	Moderate	Y
	Walcott, IA	106	4	TC	Normal	Moderate	Y
	Lexington, IL	115	4	TC + AI 400	Unexpected	High	Y
	Wyoming, IL	115	4	TC	Unexpected	Moderate	Y
	Peterson, MN	96	4	TC	Unexpected	High	Y
	Springfield, MN	96	4	TC	Unexpected	Low	Y
	Lanesboro, MN	96	4	TC	Unexpected	High	N
	Scottsbluff, NE	96	4	TC	Normal	High	Y
	Colman, SD	96	4	TC	Unexpected	Low	Y
	Arlington, WI	96	4	TC	Unexpected	High	Y

a Relative maturity of the corn hybrids planted at each site.

b Trap crop (TC) consisted of mixed maturity corn planted the previous year or a mix of corn and pumpkins. Artificial infestation (AI) occurred at V2–V5 at 400 or 800 eggs per 0.305 m of row.

c Trials were categorized post hoc for local rootworm population tolerance to hybrids only expressing Cry3 proteins (either Cry3Bb1 or mCry3A).

d Trials were assigned one of the three feeding pressure categories based on non-Bt node injury scale (NIS) ratings: high (>2 NIS), moderate (1 to < 2 NIS), or low (<1 NIS).

e Yield was not collected at all locations. Sites with Y = yield collected, sites with N = no yield collected.

**Table 2:** Treatments used in studies

Trt no.	Product name	Proteins targeting corn rootworm	Events targeting corn rootworm	Soil insecticide
1	Non-Bt^a^	None	None	None
2	Non-Bt^a^	None	None	Tefluthrin^b^
3	Herculex XTRA^c^	Cry34Ab1/Cry35Ab1	DAS-59122-7	None
4	Herculex XTRA^c^	Cry34Ab1/Cry35Ab1	DAS-59122-7	Tefluthrin^b^
5	SmartStax^d^	Cry34Ab1/Cry35Ab1	DAS-59122-7	None
		Cry3Bb1	MON88017	
6	SmartStax^d^	Cry34Ab1/Cry35Ab1	DAS-59122-7	Tefluthrin^b^
		Cry3Bb1	MON88017	

a Contained event NK603 conferring glyphosate-tolerance (Monsanto Co.).

b Force 3G at 185 g active ingredient per ha, Sygnenta Crop Protection LLC.

c Dow AgroSciences LLC.

d Dow AgroSciences LLC and Monsanto Co.

All trials were planted on areas that had been a trap crop (late-planted corn and pumpkins, *Cucurbita pepo* L.) the previous year, to attract ovipositing female corn rootworm beetles. Sites generally contained a mix of western and northern corn rootworms. Based on visual estimations of adult beetles, most sites were dominated by western corn rootworms (with western corn rootworms comprising 90–95% of the population); however, two sites in southeastern Minnesota reported northern corn rootworms accounting for 15 and 21% of the total corn rootworm population (based on adult sticky trap counts). Several locations were also artificially infested with western corn rootworm eggs at a rate of ∼1,300 or 2,600 eggs per meter at the V2 to V5 stage of corn (Ritchie et. al. 1993) during the trial year (Table 1). Artificial infestation was used to increase pressure on perceived lower pressure sites that were also believed to only contain Bt-susceptible natural populations. Artificially infested western corn rootworm eggs (Sutter and Branson 1980) were from nondiapausing insecticide- and Bt-susceptible colonies from either Crop Characteristics, Inc. (Farmington, MN) or French Agricultural Research, Inc. (Lamberton, MN; Table 1).

Treatments were arranged in a randomized complete block design at each trial location, with three to four replications depending on the available trap crop space from the previous season (Table 1). Plots were 3 by 6 m (four rows on 76.2-cm centers). All trials were maintained using standard agronomic practices for optimal crop productivity, which varied slightly across trials (herbicide application rates, nutrient applications, and tillage type). In mid- to late-July of each trial year, at approximately the VT to R2 corn growth stage (Ritchie et. al. 1993), five randomly selected plants were dug in row 1 or 4 from each plot. Corn rootworm injury to each associated root mass was then assigned a rating from 0 to 3 using the Iowa State University node-injury scale (NIS; Oleson et al. 2005). Yield was collected on the center rows (rows 2 and 3) at sites where harvest equipment was available (various small plot combines; Table 1). Yield data were corrected for moisture at 15% and converted to kg grain per ha for analysis.

Trials were categorized post hoc for overall rootworm feeding pressure and local rootworm population tolerance to Cry3-expressing plants (either Cry3Bb1 or mCry3A). Trials were assigned one of the three root-feeding pressure groupings based on the NIS for the non-Bt control treatment; low pressure was defined as an NIS of 0 to less than 1, moderate pressure as 1 to less than a 2 on the NIS, and high pressure as 2 to 3 on the NIS (Table 1). For each trial, rootworm populations were characterized as causing unexpected damage to the Cry3-containing hybrids if NIS ratings of 1 or higher were observed on adjacent Cry3Bb1 (MON 88017, Monsanto Co.) and mCry3A (event MIR 604, Syngenta Crop Protection LLC) hybrids within the same trial year, or from results acquired in a previous year (Table 1). Unexpected damage classifications were further corroborated based on field population history for each trial.

Mean NIS ratings and grain yield for each of the resulting six Cry3Bb1-expressing root injury-by-feeding pressure scenarios were analyzed separately using a mixed model analysis of variance (ANOVA; PROC MIXED; SAS Institute Inc. 2011). Each scenario was analyzed separately, as Cry3-expressing corn root injury-by-treatment interactions were inherent to the test of treatment performance, and feeding pressure was likely partially confounded with root injury to Cry3Bb1-expressing hybrids. Therefore, only descriptive statistics are provided for summaries across all trials at the treatment level. The large data set satisfied requirements for ANOVA that are typically unmet for NIS ratings when treatments include unprotected and protected corn roots under significant feeding pressure (Prasifka et al. 2013). Treatment was considered a fixed effect; and year, location, replicate (block) within location, location-by-year, and location-by-treatment interactions were considered random. Paired contrasts were performed between treatments and letter assignments were made following application of Fisher’s protected test (LSD; α = 0.05). Descriptive statistics were calculated using PROC MEANS (SAS Institute Inc. 2011).

## Results

The data presented a mixture of sites categorized as having unexpected Cry3-expressing corn root injury (21 trials) and sites without unexpected root injury to hybrids expressing Cry3 only (22 trials). As a determination of feeding pressure, NIS ratings in the non-Bt treatment included 15 trials reporting greater than two nodes consumed (high feeding pressure), 14 trials reporting one to less than two nodes consumed (moderate feeding pressure), and 14 trials reporting less than one node consumed (low feeding pressure; Table 1). Significant treatment effects were observed in all feeding pressure scenarios for both root injury and yield (Table 3).

**Table 3:** Summary of statistical results for 43 trials subjected to analysis of variance

Injury level to Cry3-expressing corn roots	Root-feeding pressure on non-Bt corn^a^	Node injury			Yield		
		*F*	df	*P*^b^	*F*	df	*P*^b^
Unexpected (>1.0)	High	150.16	51.1	< 0.001	15.39	45.9	< 0.001
	Moderate	45.12	25.2	< 0.001	1.55	25.1	0.209
	Low	12.97	20	< 0.001	1.47	19.5	0.245
Normal (<1.0)	High	104.42	110	< 0.001	1.93	15	0.148
	Moderate	75.24	24.8	< 0.001	4.88	26.1	0.003
	Low	7.73	48.2	< 0.001	5.89	25.5	0.001

a Trials were assigned one of the three feeding pressure categories based on non-Bt node injury scale (NIS) ratings: high (>2 NIS), moderate (1 to < 2 NIS), or low (<1 NIS).

b Fisher’s LSD.

**Fig. 1:**
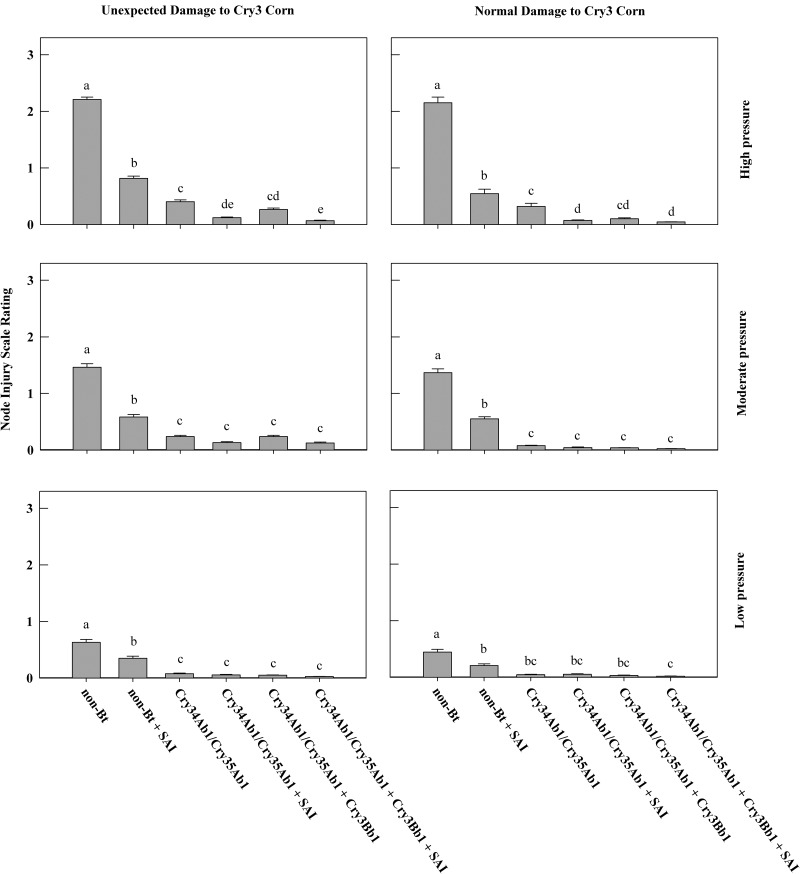
Mean node injury scale (NIS) rating ± standard error of corn hybrids with or without soil-applied insecticide (SAI), presented by level of corn rootworm injury to Cry3-expressing corn (normal or unexpected) and under high (>2 NIS), moderate (1–2 NIS), or low (<1 NIS) root feeding pressure. NIS ratings with the same letter do not differ significantly (LSD, α = 0.05).

**Fig. 2:**
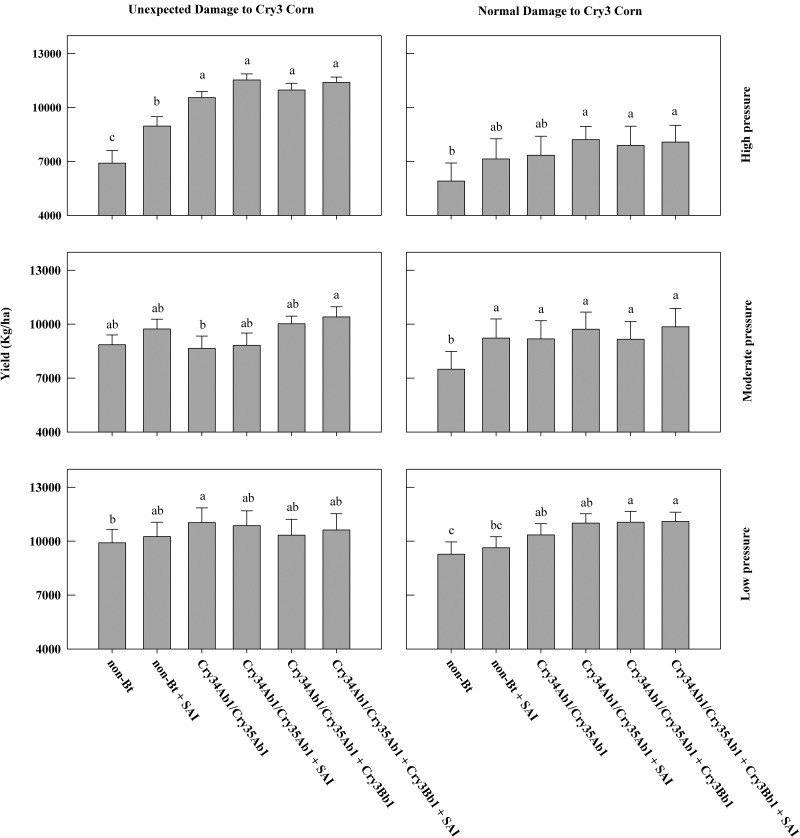
Mean grain yield (kg/ha) ± standard error of corn hybrids with or without soil-applied insecticide (SAI), presented by level of corn rootworm injury to Cry3-expressing corn (normal or unexpected) and under high (>2 NIS), moderate (1–2 NIS), or low (<1 NIS) root feeding pressure. Yield with the same letter do not differ significantly (LSD, α = 0.05).

At high-pressure sites with unexpected root injury to Cry3-expressing hybrids (nine trials), Cry34Ab1/Cry35Ab1 alone had an average NIS of 0.4 ± 0.03 (mean ± SEM) nodes of roots consumed, which was significantly lower than the non-Bt (NIS = 2.21 ± 0.04) or non-Bt + soil-applied insecticide (NIS = 0.82 ± 0.04) treatments (Fig. 1). The addition of SAI to both Cry34Ab1/Cry35Ab1 alone (without soil insecticide NIS = 0.4 ± 0.03, with soil insecticide NIS = 0.12 ± 0.01) and Cry34Ab1/Cry35Ab1 pyramided with Cry3Bb1 (without soil insecticide NIS = 0.25 ± 0.03, with soil insecticide NIS = 0.07 ± 0.01) significantly reduced NIS compared to the traits alone (Fig. 1). At high feeding pressure sites with unexpected root injury to Cry3-expressing hybrids, the additional root protection provided by the SAI in conjunction with hybrids expressing Cry34Ab1/Cry35Ab1 alone and Cry34Ab1/Cry35Ab1 pyramided with Cry3Bb1 did not result in significant increases in yield compared to traits without SAI (Fig. 2).

At sites with high feeding pressure to non-Bt hybrids but without unexpected root injury to Cry3-expressing corn (six trials), plants expressing Cry34Ab1/Cry35Ab1 (NIS of 0.32 ± 0.06 for Cry34Ab1/Cry35Ab1 alone and 0.1 ± 0.02 for Cry34Ab1/Cry35Ab1 pyramided with Cry3Bb1) had significantly lower root damage than the non-Bt (NIS = 2.15 ± 0.1) or non-Bt + SAI (NIS = 0.55 ± 0.08) treatments. The addition of SAI to Cry34Ab1/Cry35Ab1 alone (NIS = 0.7 ± 0.01) significantly reduced NIS ratings compared to the Bt trait alone. There was no significant increase in root protection when SAI was added to the Cry34Ab1/Cry35Ab1 alone or Cry34Ab1/Cry35Ab1 pyramided with Cry3Bb1 (NIS = 0.05 ± 0.01) under this scenario (Fig. 1.). The addition of SAI to either Cry34Ab1/Cry35Ab1 pyramided with Cry3Bb1 or Cry34Ab1/Cry35Ab1 alone did not result in significant increases in yield (Fig. 2).

At moderate feeding pressure sites with unexpected root injury to Cry3Bb1-expressing corn (seven trials), plants expressing Cry34Ab1/Cry35Ab1 had significantly lower root feeding (NIS of 0.24 ± 0.02 for Cry34Ab1/Cry35Ab1 alone and 0.24 ± 0.02 for Cry34Ab1/Cry35Ab1 pyramided with Cry3Bb1 hybrids) than either the non-Bt (NIS = 1.46 ± 0.06) or non-Bt + SAI (NIS = 0.58 ± 0.05) treatments (Fig. 1). The addition of SAI to either Cry34Ab1/Cry35Ab1 pyramided with Cry3Bb1 or Cry34Ab1/Cry35Ab1 alone did not significantly reduce NIS ratings compared with the traits alone (Fig.1). In addition, SAI applied to either Cry34Ab1/Cry35Ab1 pyramided with Cry3Bb1 and Cry34Ab1/Cry35Ab1 alone did not result in significant increases in yield (Fig. 2).

At moderate feeding pressure sites without unexpected root injury to Cry3Bb1-expressing corn (seven trials), plants expressing Cry34Ab1/Cry35Ab1 had significantly lower root feeding (NIS of 0.08 ± 0.01 for Cry34Ab1/Cry35Ab1 alone and 0.04 ± 0.01 for Cry34Ab1/Cry35Ab1 pyramided with Cry3Bb1) than either the non-Bt (NIS = 1.37 ± 0.07) or non-Bt + SAI (NIS = 0.55 ± 0.04) treatments (Fig. 1). Yield from the non-Bt + SAI was not significantly different from Cry34Ab1/Cry35Ab1 alone with or without SAI. There was no significant reduction in root injury when the soil-applied insecticide was added to either Cry34Ab1/Cry35Ab1 pyramided with Cry3Bb1 or Cry34Ab1/Cry35Ab1 alone (Fig. 1). The use of SAI with either Cry34Ab1/Cry35Ab1 pyramided with Cry3Bb1 or Cry34Ab1/Cry35Ab1 alone did not result in significant increases in yield (Fig. 2).

At low feeding pressure sites with unexpected root injury to Cry3Bb1-expressing corn (five trials), plants expressing Cry34Ab1/Cry35Ab1 had significantly lower root feeding (NIS of 0.08 ± 0.01 for Cry34Ab1/Cry35Ab1 alone and 0.05 ± 0.01 for Cry34Ab1/Cry35Ab1 pyramided with Cry3Bb1) than either the non-Bt (NIS = 0.63 ± 0.05) or non-Bt + SAI (NIS = 0.35 ± 0.04) treatments (Fig. 1). The addition of SAI to either Cry34Ab1/Cry35Ab1 alone or Cry34Ab1/Cry35Ab1 pyramided with Cry3Bb1 did not significantly reduce NIS or increase yields of the traits alone (Figs. 1 and 2).

At low-pressure sites without unexpected root injury to Cry3Bb1-expressing corn (nine trials), plants expressing Cry34Ab1/Cry35Ab1 had significantly lower root feeding (NIS of 0.05 ± 0.01 for Cry34Ab1/Cry35Ab1 alone and 0.03 ± 0.01 for Cry34Ab1/Cry35Ab1 pyramided with Cry3Bb1) than the non-Bt treatment (NIS = 0.45 ± 0.05; Fig. 1). The addition of SAI to either Cry34Ab1/Cry35Ab1 alone (NIS = 0.05 ± 0.01) and Cry34Ab1/Cry35Ab1 pyramided with Cry3Bb1 (NIS = 0.02 ± 0.01) did not significantly reduce NIS or increase yields of the traits alone (Figs. 1 and 2). Cry34Ab1/Cry35Ab1 pyramided with Cry3Bb1 showed a significant increase in yield compared to the non-Bt + SAI treatment (Fig. 2).

Over all feeding pressures, the addition of a soil insecticide to non-Bt corn resulted in a significant reduction in NIS ratings compared to the non-Bt alone across both populations of rootworm (normal damage to Cry 3 Corn, and unexpected damage to Cry 3 Corn; Fig. 1). Both Cry34Ab1/Cry35Ab1 pyramided with Cry3Bb1 and Cry34Ab1/Cry35Ab1 alone resulted in a significant reduction in NIS compared with the non-Bt treatment across both populations of rootworm (normal damage to Cry 3 Corn, and unexpected damage to Cry 3 Corn; Fig. 1). The addition of SAI to Cry34Ab1/Cry35Ab1 alone reduced NIS ratings significantly only under situations of high feeding pressure with or without unexpected root injury to Cry3-expressing corn (Fig. 1). The addition of SAI to Cry34Ab1/Cry35Ab1 pyramided with Cry3Bb1 reduced NIS ratings significantly only under situations of high feeding pressure and unexpected root injury to Cry3-expressing corn (Fig. 1). Yield gains from the use of SAIs on hybrids with Bt traits were not significant in this study (Fig. 2). However, there is a very consistent trend where numerical increases in yield tracked the reduction in root injury from the addition of SAI at all six pressure and population groupings (Fig. 2).

## Discussion

This extensive study was a field investigation conducted over three years and included a total of 43 trials at 31 unique locations across the northern Corn Belt. This study examined the effects of rootworm Bt traits and soil-applied insecticides on root protection and grain yield. This study is unique in its comparison of field trial results on field populations of western corn rootworm where Cry3 trait-protected plants did not provide adequate root protection. We believe this to be the first field research to compare the effect of combining soil insecticides and corn rootworm-targeting Bt proteins on populations of western corn rootworm where Cry3 Bt proteins are not entirely protecting corn roots.

Although there are several options available for management of corn rootworm populations, crop rotation, soil- or seed-applied insecticides, and Bt hybrids are the most commonly adopted (Gray et al 2009). These data showed that significant root protection from the application of soil insecticides to a non-Bt expressing hybrid was achieved at sites where excessive root damage was occurring on Cry3-protected plants (Fig. 1). However, root protection from Cry34Ab1/Cry35Ab1 expressing hybrids either as a single trait in Herculex XTRA or in the pyramided with the Cry3Bb1 trait package offered by SmartStax proved to be superior to non-Bt hybrids protected by soil insecticides on all populations of corn rootworm except when feeding pressure was less than one node (<1 NIS), and susceptible to Cry3Bb1 (Fig. 1).

Both SmartStax and Herculex XTRA trait-protected plants expressing Cry34Ab1/Cry35Ab1 had lower root injury than plants whose only source of protection was soil-applied insecticide when feeding exceeded >1 node of injury (Fig. 1). Field evaluations conducted prior to the development of Cry3 resistance in populations of western corn rootworm found that SmartStax hybrids provided superior root protection compared to either Cry3Bb1 or Cry34Ab1/Cry35Ab1 single trait hybrids (Prasifka et al. 2013, Head et al. 2014). The results of this research showed that pyramided technology continues to provide very high levels of root protection against field populations of corn rootworms, although not significantly better than Cry34Ab1/Cry35Ab1 hybrids (Fig. 1). Results from individual trials showed significant root protection from pyramiding Cry34Ab1/Cry35Ab1 with a Cry3 protein (SmartStax) because it theoretically ensures that any Cry3-susceptible individuals are controlled. This will be especially important if a given population has been exposed to Cry34Ab1/Cry35Ab1-only trait technologies for a significant period.

This research also showed minimal value in adding soil insecticides even at sites where plants are not adequately protected by Cry3 traits if the hybrids are expressing the Cry34Ab1/Cry35Ab1 trait. The root protection provided by SmartStax alone prevented root feeding from exceeding an NIS rating of 0.25, which placed SmartStax in the highest yielding group for each of the feeding pressure and Cry3 injury groupings (Fig. 1). In most of the scenarios examined, application of soil insecticides to Bt-protected plants did not provide significant additional root protection except when NIS ratings exceeded 2.0 for the non-Bt treatments. These results are consistent with other published research on Cry3Bb1 susceptible populations (Petzold-Maxwell et al. 2013, Estes et al. 2015). In this high feeding pressure scenario, there was an improvement in root protection from the addition of a soil insecticide on Cry34Ab1/Cry35Ab1 single trait-protected plants. The addition of a soil insecticide only benefitted pyramided technology when the NIS rating exceeded a 2.0 for the non-Bt treatment. These results are comparable with other recent work applying meta-analysis to data on Bt-susceptible populations (Tinsley et al. 2016) and research evaluating the mCry3A + eCry3.1Ab pyramid with the addition of soil-applied insecticides (Frank et al. 2015). However, growers are reluctant to risk yield loss due to corn rootworm injury, and with reported yield losses of 15 to 17% for every node of roots lost to larval feeding (Dun et al. 2010; Tinsley et al. 2013), some growers have elected to apply soil insecticides in conjunction with Bt trait protected hybrids. The present research indicates that prophylactic soil insecticide use may provide root protection and economic benefits only under limited conditions.

It is anticipated that hybrids expressing Cry34Ab1/Cry35Ab1 will be utilized on more acres in pyramided products resulting in increased selection pressure on corn rootworm populations to develop resistance to this important plant protection trait. Under the expectation of eventual selection or resistance evolution, an increase in Cry34Ab1/Cry35Ab1 product adoption accentuates the need for growers to follow best management practices for corn rootworm that reduce insect feeding pressure. Gassmann et al. (2016) recently described a field population with “incomplete resistance” to Cry34Ab1/Cry35Ab1. Despite the high feeding pressure at most of the trial sites, Cry34Ab1/Cry35Ab1-expressing hybrids continued to provide an expected level of control below one node consumed on single trait technology (Herculex XTRA) and below 0.5 on the NIS for the pyramided technology (SmartStax) technologies in all 43 site-years of this study.

This research indicates that a soil insecticide added to a single Bt-protected technology is only beneficial when corn rootworm populations are high enough that larval feeding would result in root injury above two nodes consumed. However, this could require NIS evaluations, adult monitoring, and egg sampling the year prior to the corn crop to verify effectiveness of Bt traits and sufficient insect pressure exists to justify the added expense of a soil insecticide. In fields where larval feeding would result in root injury below 2 on the NIS, there is no detected benefit of including a soil insecticide with a pyramided trait package.
